# Adaptive fuzzy deep learning with multimodal sensor fusion for enhanced plant disease detection

**DOI:** 10.1038/s41598-026-50281-z

**Published:** 2026-05-16

**Authors:** Sangeetha S.K.B, Royappan Lotus, Deeba K, Benjula Anbu Malar M.B, Basu Dev Shivahare, Sandeep Kumar Mathivanan

**Affiliations:** 1 Manipal Institute of Technology Bengaluru, Manipal Academy of Higher Education, Manipal, India; 2 Department of Computer Science and Engineering, Vel Tech Rangarajan Dr.Sagunthala R&D Institute of Science and Technology, Avadi, Chennai, Tamil Nadu India; 3 School of Computer Science and Applications, REVA University, Bangalore, India; 4 School of Computer Science Engineering and Information Systems, Vellore Institute of Technology, Vellore, Tamil Nadu India; 5 School of Computing Science and Engineering, Galgotias University, Greater Noida, 203201 India; 6 Department of Computer Science & Engineering- AI & ML, KG Reddy College of Engineering and Technology, Hyderabad, Telangana, 501504 India

**Keywords:** Plant disease detection, Deep learning, Fuzzy logic, Multimodal data fusion, Deep neural networks, Computational biology and bioinformatics, Engineering, Mathematics and computing, Plant sciences

## Abstract

Timely and accurate plant disease detection is important for enhancing agricultural productivity and promoting sustainability. The study introduces Multimodal Adaptive Fuzzy-based Deep Neural Network (MAF-DNN) for classification of plant diseases. The proposed method combines fuzzy logic with multimodal data fusion to effectively address the complex interactions and uncertainties in agricultural datasets. The MAF-DNN employs a robust adaptive fuzzy framework with dynamic rule optimization and integrates Hyperspectral Imaging Data (HID) with RGB imaging data to acquire detailed spectral information and high-resolution visual cues for disease classification. The multimodal fusion enhances the model’s ability to capture intricate patterns that relate to plant health, improving the accuracy of disease classification. The experimental results showed that the MAF-DNN outperforms traditional models by achieving an accuracy of 97.8%, precision of 96.5%, recall of 98.2%, and F1-score of 97.3%. Additionally, the adaptive design reduces computational overhead, increases efficiency, and improves scalability for large-scale agricultural applications. The MAF-DNN represents a significant advancement in plant disease classification and provides a robust and efficient solution for precision agriculture.

## Introduction

 Plant disease detection forms a very significant area because of its strong impact upon agricultural productivity, food security, and sustainable farming practices^1^. For the backbone of the entire food supply in the world, this is a critical concern because there are many diseases caused to plants by various pathogens from fungi, bacteria, viruses, etc^2^. These diseases result in huge crop losses; hence, it threatens farmer’s livelihood and increase starvation in the world. With a growing global population and limited arable land, the health and productivity of crops have never been more important^3^. Timely and accurate plant disease detection plays a very important role in minimizing losses and ensuring sustainable agriculture with an uninterrupted food supply chain. Historically, plant disease diagnosis relied on manual inspection of the crops by agricultural experts and farmers. Symptoms, such as discoloration, spots, lesions, wilting, or deformities, are visually examined to diagnose the diseases. This method has been effective to some extent but is inherently time-consuming, labor-intensive, and prone to human error^4^. The accuracy of diagnosis relied more on the expertise of the inspector, who was not always accessible, especially in remote or underdeveloped regions. These factors showed that there was a greater need for more efficient and objective methods for disease detection^5^. Plant disease detection was revolutionized by the advent of technology^6^. Digital image analysis was one of the early applications of image processing, which allowed one to analyze digital images and detect and quantify disease symptoms. These methods were compared with manual inspection and have significant advantages in terms of producing objective, reproducible, and quantifiable results^7^. For example, digital image analysis might detect subtle variations in color, texture, and lesion shape that are not easy to visualize by the human eye^8^. Building on these advances, it was machine learning that allowed the development of supervised learning algorithms based on labeled datasets of both diseased and healthy images of plants^9^. Decision trees, support vector machines, and k-nearest models became popular as they also predicted the presence of a disease in new samples by using the training dataset^10^.

Despite these advances, early methods faced significant limitations. One of the primary challenges was the variability of disease symptoms across different plant species and even among cultivars of the same species. Factors such as genetic diversity, environmental conditions, and stages of infection caused symptoms to manifest differently^11^. For example, spots from a fungal infection on a leaf may differ in size, shape, and color with a specific crop and environment. Another aspect is the symptom pattern from abiotic factors such as nutrient deficiency, drought, or pesticide damage^12^. More often, it resulted in similar symptom manifestation that made correct identification rather difficult. Another major limitation of traditional models was scalability^13^. Many of these approaches were dependent on handcrafted features and required domain expertise to handpick and engineer features from raw data. The time-consuming process failed to grasp the complex, hierarchical patterns that exist in plant images^14^. As datasets grew and complexity, models ran into problems handling high-dimensional data efficiently, thereby compromising accuracy and increasing computational loads^15^. The development of deep learning, specifically Convolutional Neural Networks (CNNs), has revolutionized the detection of plant diseases^16^. CNNs are outstanding at automatically extracting hierarchical features from images, which means that they can easily identify the slight patterns and differences indicating a disease. These models have demonstrated remarkable performance in image recognition tasks, such as plant disease classification^17^. Thus, the CNNs streamlined the disease detection process by eliminating the necessity of manual feature engineering, making it more efficient and scalable. However, CNN-based approaches have their limitations^18^.

Most of the existing models are crop or disease specific and therefore cannot be applied in real-world agricultural settings where a variety of crops and diseases are present^19^. For instance, a model trained to detect rice leaf blight may not recognize wheat rust or tomato mosaic virus^20^. The lack of generalizability hinders the practical deployment of these models on farms, where mixed cropping systems are common^21^. These challenges can be addressed using the new approach called multimodal data fusion, which combines several data sources into a better representation of the problem^22^. In this regard, in plant disease detection, multimodal fusion includes combining Hyperspectral Image Data (HID) and RGB imaging. HID captures the spectral information of the targeted plant over a broad range, from beyond the visible spectrum, disclosing slight biochemical changes indicative of a disease^23^. For instance, a change in chlorophyll content or water levels, indicating of early stress, can be sensed in certain spectral bands. RGB imaging provides high-resolution visual information such as color, texture, and shape, necessary for identifying visible symptoms, such as lesions or spots^24^. By combining these modalities, multimodal fusion enables models to leverage both spectral and visual features, enhancing their ability to detect diseases accurately and robustly across diverse conditions. This approach improves generalizability, making it suitable for real-world agricultural applications^25^.

Building on these advancements, this study introduces a novel Multimodal Adaptive Fuzzy Deep Neural Network (MAF-DNN) for plant disease detection. This integrates fuzzy logic with multimodal data fusion to address the drawbacks of existing methods. The model is enhanced by adding fuzzy logic to handle the uncertainty and noise in data; in plant disease symptoms, variation is very common. This ability in modeling imprecise and ambiguous data is enhanced by the introduction of fuzzy reasoning into the neural network framework, thus making the MAF-DNN more robust and reliable. The MAF-DNN adopts a hierarchical fuzzy layer structure that optimizes fuzzy rules dynamically, according to input data. The adaptive framework would be able to handle any variability and complexity in the agricultural datasets. Further, multimodal data fusion will ensure the model uses a set of features from both HID and RGB data. Together, these innovations ensure that the MAF-DNN is a strong, efficient, and scalable solution toward plant disease detection, putting the basis for further applications in precision agriculture and sustainable agricultural practices^26,27^.

The main contribution is.

- To develop the multimodal adaptive Fuzzy Deep Neural Network (MAF-DNN), a novel architecture that integrates adaptive fuzzy logic with multimodal data fusion to address uncertainty and complexity in plant disease detection datasets.
- To implement dynamic rule optimization within the adaptive fuzzy framework to enhance the model’s ability to handle diverse and uncertain agricultural data effectively.
- To integrate hyperspectral imaging data (HID) with RGB imaging data, enabling the model to capture intricate patterns related to plant health and disease manifestations.

The introduction of MAF-DNN represents a significant advancement in the field of plant disease detection. By combining fuzzy logic, and multimodal data fusion, this approach provides a robust, accurate, and efficient solution, paving the way for future research and development in agriculture. The detailed implementation process, including data description, preprocessing, model architecture design in Sect.  2, and performance evaluation in Sect.  3, followed by conclusion in Sect.  4 is thoroughly discussed in this study.

## System methodology

### Dataset used

**Fig. 1:**
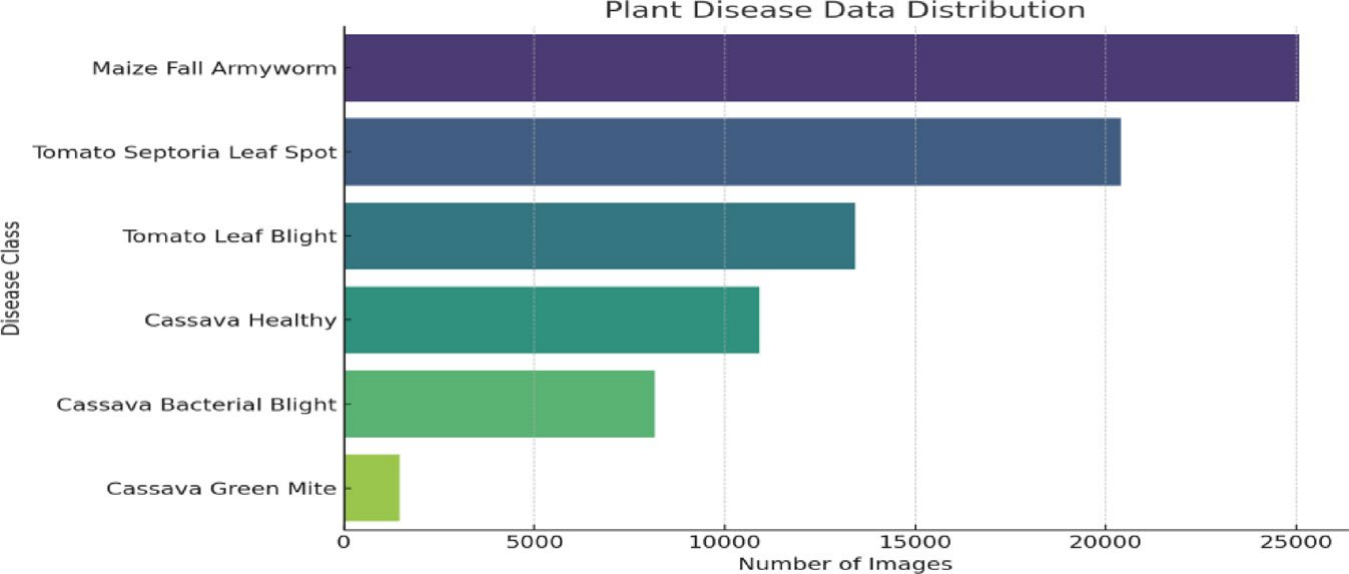
Dataset distribution.

**Fig. 2:**
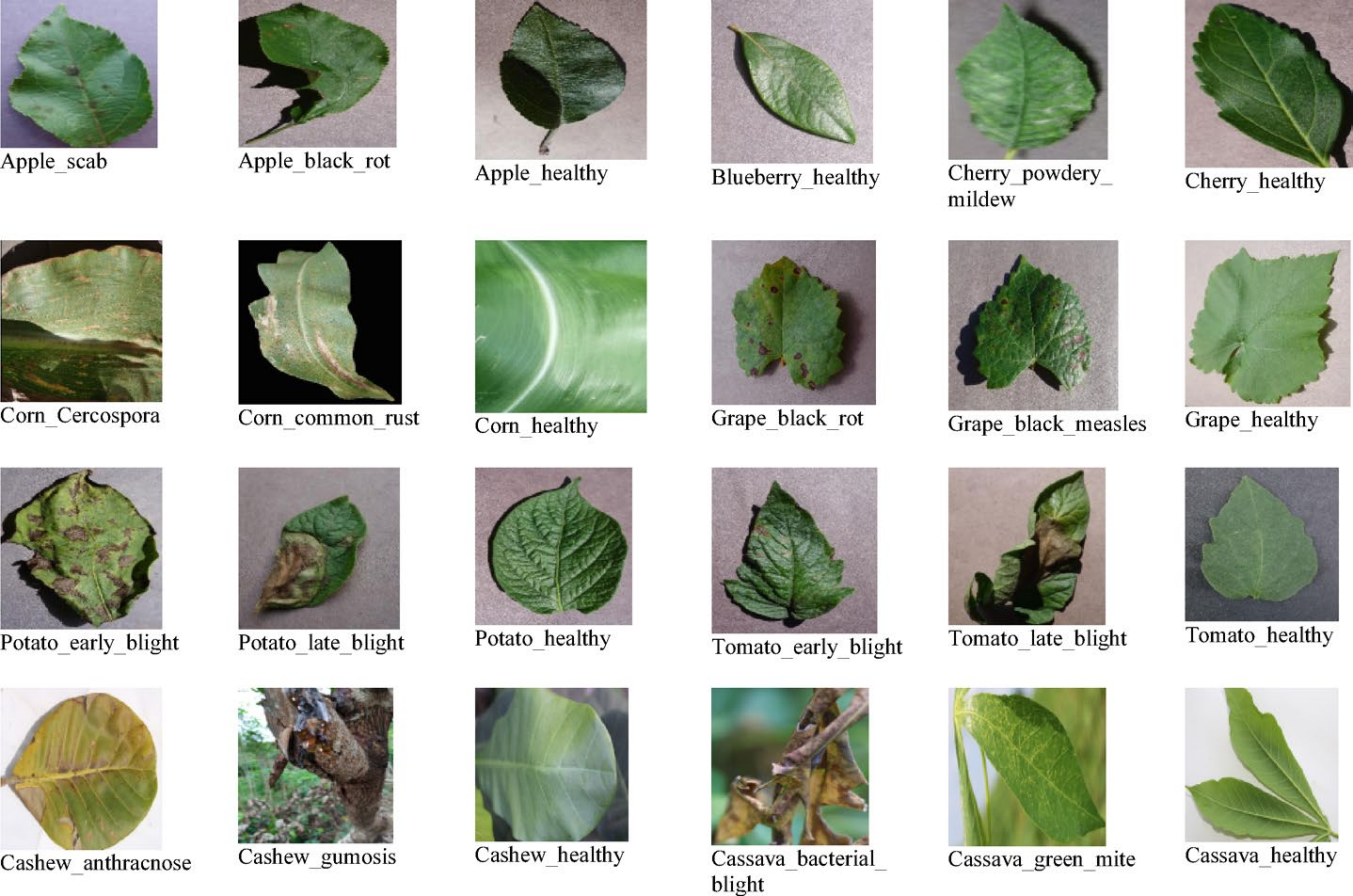
Image categories.

70,295 images from 14 plant species such as tomatoes, grapes, and potatoes comprise the New Plant Diseases Dataset(https://www.kaggle.com/datasets/vipoooool/new-plant-diseases-dataset*)*. The amount varies by species with 10,408 images of corn and 3,213 images of raspberries. To provide a well-rounded set to train machine learning algorithms, images are annotated by plant species as well as disease types, bacterial spot and blight. With 24,881 images taken in Ghana, the CCMT Plant Disease Dataset(https://www.kaggle.com/datasets/rahimanshu/ccmt-plant-disease-dataset*)* has a focus on four crops: cashew, cassava, tomato, and maize. The images are categorized into 22 classes and have pests like the fall armyworm and diseases like powdery mildew. The custom code used to develop and evaluate the proposed Multimodal Adaptive Fuzzy Deep Neural Network (MAF-DNN) framework is publicly available at: https://github.com/skbsangeetha/MAF-DNN-Plant-disease-classification The repository includes all core components required to reproduce the results reported in this study, including data preprocessing, feature extraction (edge, texture, and color features), fuzzy logic-based feature transformation, adaptive multimodal fusion, and deep neural network training and evaluation. Figure 1 depicts the data distribution. Figure 2 shows the sample images.

**Fig. 3:**
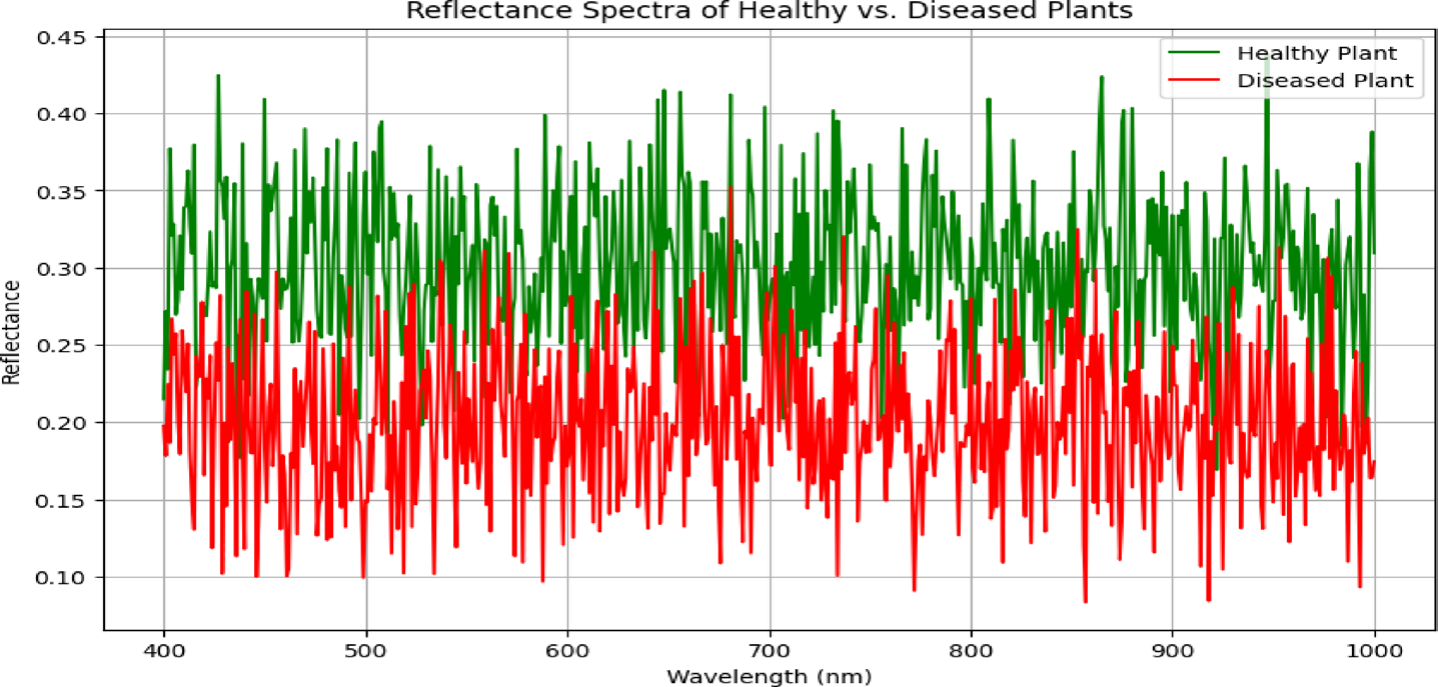
Reflectance of healthy and stressed plants.

In hyperspectral imaging, the x-axis represents the wavelength in nanometers, typically from 400 nm to 1000 nm, where it covers the visible to near-infrared spectrum. The y-axis depicts reflectance value, a quantification of the amount of light reflected from the surface by the plant. The green line represents reflectance spectra of a healthy plant, and the red line represents reflectance spectra of an unhealthy plant, as illustrated in Fig. 3.

In the 400–500 nm range (blue to green), healthy plants exhibit a low reflectance because of the strong absorption by chlorophyll pigments. In the case of diseased plants, increased reflectance is likely in this region due to chlorophyll degradation. Between 500 and 600 nm, this range is sensitive to chlorophyll content. Healthy plants have a characteristic absorption peak at around 550 nm. The diseased plant may have its absorption pattern changed, reflecting changes in chlorophyll content. In the red range of 600–700 nm, healthy plants have a peak absorption around 675 nm due to chlorophyll absorption. Diseased plants may absorb less in this range, thereby showing increased reflectance. Between 700 and 1000 nm (near-infrared), healthy plants reflect highly in this region due to the internal structure of the leaf and water content. Diseased plants may reflect less in this range, indicating changes in the structure of the leaf or water content. Hyperspectral imaging can detect subtle changes in plant physiology by analyzing these specific wavelength regions, allowing for early identification of diseases before visible symptoms appear.

### Data preprocessing

In preprocessing, the first step is image resizing, which ensures all images are resized to a uniform size of 224 × 224 pixels, a standard resolution used in image classification tasks. Normalization of pixel values is then performed, scaling the values to the range [0,1] to enhance uniformity and facilitate faster convergence during training. Data augmentation techniques, including random rotations (± 20 degrees), horizontal flipping, and brightness adjustments, are applied to increase dataset diversity and reduce overfitting. Augmentation is implemented using libraries like TensorFlow’s ImageDataGenerator or PyTorch’s transforms. To optimize memory usage and training efficiency, images are grouped into 32 batches. The dataset is also shuffled to prevent the model from learning unintended sequential patterns. Class imbalance is addressed through class weighting, ensuring that minority classes receive appropriate attention during training. Additionally, noise and outliers, such as blurry images or mislabeled data, are minimized through quality enhancement techniques and manual review. Finally, the data is split into training (80%), validation (10%), and testing (10%) subsets to ensure robust evaluation of the model’s performance. Tables 1 and 2 illustrate the dataset features before and after preprocessing, respectively.

**Table 1:** Before preprocessing - dimension factor.

Image ID	Plant/disease	Dimensions	Pixel range
1	Apple_scab	800 × 600	[0, 255]
2	Apple_black_rot	1024 × 768	[0, 255]
3	Tomato_early_blight	640 × 480	[0, 255]
4	Grape_black_rot	800 × 600	[0, 255]
5	Corn_common_rust	1280 × 960	[0, 255]
6	Potato_early_blight	800 × 600	[0, 255]
7	Cashew_anthracnose	1024 × 768	[0, 255]
8	Cassava_bacterial_blight	640 × 480	[0, 255]
9	Maize_fall_armyworm	800 × 600	[0, 255]
10	Tomato_late_blight	1024 × 768	[0, 255]

**Table 2:** After preprocessing - dimension factor.

Image ID	Plant/disease	Dimensions	Pixel range
1	Apple_scab	224 × 224	[0, 1]
2	Apple_black_rot	224 × 224	[0, 1]
3	Tomato_early_blight	224 × 224	[0, 1]
4	Grape_black_rot	224 × 224	[0, 1]
5	Corn_common_rust	224 × 224	[0, 1]
6	Potato_early_blight	224 × 224	[0, 1]
7	Cashew_anthracnose	224 × 224	[0, 1]
8	Cassava_bacterial_blight	224 × 224	[0, 1]
9	Maize_fall_armyworm	224 × 224	[0, 1]
10	Tomato_late_blight	224 × 224	[0, 1]

**Table 3:** Edge detection and texture features.

Image ID	Plant/disease	Edge features	Texture features
1	Apple_scab	[0.75, 0.82, …]	[0.45, 0.38, …]
2	Apple_black_rot	[0.68, 0.75, …]	[0.37, 0.44, …]
3	Tomato_early_blight	[0.82, 0.89, …]	[0.52, 0.46, …]
4	Grape_black_rot	[0.76, 0.83, …]	[0.47, 0.39, …]
5	Corn_common_rust	[0.69, 0.76, …]	[0.38, 0.45, …]
6	Potato_early_blight	[0.83, 0.90, …]	[0.53, 0.47, …]
7	Cashew_anthracnose	[0.76, 0.83, …]	[0.48, 0.40, …]
8	Cassava_bacterial_blight	[0.68, 0.75, …]	[0.39, 0.46, …]
9	Maize_fall_armyworm	[0.84, 0.91, …]	[0.54, 0.48, …]
10	Tomato_late_blight	[0.77, 0.84, …]	[0.46, 0.39, …]

**Table 4:** Color histograms.

Image ID	Plant/disease	Red histogram	Green histogram	Blue histogram
1	Apple_scab	[0.25, 0.33, …]	[0.36, 0.45, …]	[0.15, 0.22, …]
2	Apple_black_rot	[0.31, 0.40, …]	[0.42, 0.50, …]	[0.21, 0.28, …]
3	Tomato_early_blight	[0.39, 0.48, …]	[0.51, 0.60, …]	[0.31, 0.38, …]
4	Grape_black_rot	[0.47, 0.56, …]	[0.58, 0.67, …]	[0.41, 0.48, …]
5	Corn_common_rust	[0.55, 0.64, …]	[0.65, 0.74, …]	[0.51, 0.58, …]
6	Potato_early_blight	[0.63, 0.72, …]	[0.72, 0.81, …]	[0.61, 0.68, …]
7	Cashew_anthracnose	[0.71, 0.80, …]	[0.79, 0.88, …]	[0.71, 0.78, …]
8	Cassava_bacterial_blight	[0.79, 0.88, …]	[0.87, 0.96, …]	[0.81, 0.88, …]
9	Maize_fall_armyworm	[0.87, 0.96, …]	[0.95, 1.04, …]	[0.91, 0.98, …]
10	Tomato_late_blight	[0.94, 1.03, …]	[1.02, 1.11, …]	[0.98, 1.05, …]

### Feature extraction

The feature extraction techniques employed in plant disease identification are edge detection, texture analysis, computation of color histograms, and integration of HID features (Tables 3 and 4). The detection of edges using the Canny edge detector is critical for marking lesion borders on the leaves of plants that usually signify a particular disease. In smoothing an image, non-noise details are removed from the image by performing intensity gradient computation and suppressing values that are non-maximum values to keep track of the highest significance edges. It further refines the edges determined by double thresholding to capture lesion boundaries to be quantified into numerical descriptors important for disease differentiation. In texture analysis, the Gray-Level Co-occurrence Matrix (GLCM) can be used for capturing spatial relations between pixel pairs.

Features such as contrast, correlation, energy, and homogeneity derived from the GLCM give an insight into the texture characteristics of lesions. Fungal diseases often present as being rough and irregular, such as diseases caused by rust or powdery mildew, while bacterial diseases like bacterial wilt have relatively smoother textures. Texture features thus distinguish different plant diseases based on visual patterns. Color histogram computation examines the distribution of pixel intensities across RGB channels, providing a quantitative description of the color content of an image. This is very useful for diseases that can be identified based on color changes, such as reddish-brown lesions in rust or yellowing leaves in bright. Histograms of healthy and diseased samples can then be compared to identify distinct color patterns characteristic of specific diseases. In addition, spectral or intensity fluctuations are not visually represented in EDGE, texture, and color analyses and are well characterized by HID feature representations.

For preprocessing, data from HID are normalized and scaled similarly to other datasets and then brought into the main feature extraction module. In the model, inputting HID feature data increases detection of minor features of lesions because it adds yet another layer to the diagnostic feature set. These features are fused with edge, texture, and color features to form a comprehensive dataset (Table 5), which serves as the input for the disease classification model. Inclusion of these multivariate features, such as edge details, texture patterns, color distributions, and high-intensity variations in the proposed model results in the robust and accurate presentation of plant diseases. The above fusion of the features enhance the reliability and applicability of the disease diagnosis system.

### Framework overview

The MAF-DNN (Multimodal Adaptive Fuzzy Deep Neural Network) framework is designed for robust plant disease detection using multimodal data, including hyperspectral (HID) and RGB images. As shown in Fig. 5, the framework integrates advanced feature extraction, adaptive fusion, and deep learning techniques to improve diagnostic accuracy and reliability. Key processes include extracting fuzzy features, adaptively combining multimodal data, and leveraging a specialized DNN architecture for precise classification.

Contribution 1: Adaptive feature fusion (Fig. 6).

An innovative adaptive feature fusion mechanism combines RGB and HID features dynamically. Adaptive weights are calculated using a function based on feature variances, ensuring optimal integration of complementary information from both modalities. This process enhances the model’s ability to capture subtle disease markers, contributing to higher detection accuracy.

Contribution 2: Custom deep neural network architecture (Fig. 7).

A tailored DNN architecture is implemented, featuring layers optimized for disease classification. The network balances complexity and efficiency, enabling it to process fused features effectively. The model achieves impressive performance metrics, including high precision, recall, and reduced error rates, demonstrating its superiority over traditional methods.

**Table 5:** Combined features.

Image ID	Plant/disease	Edge features	Texture features	Red histogram	Green histogram	Blue histogram
1	Apple_scab	[0.75, 0.82, …]	[0.45, 0.38, …]	[0.25, 0.33, …]	[0.36, 0.45, …]	[0.15, 0.22, …]
2	Apple_black_rot	[0.68, 0.75, …]	[0.37, 0.44, …]	[0.31, 0.40, …]	[0.42, 0.50, …]	[0.21, 0.28, …]
3	Tomato_early_blight	[0.82, 0.89, …]	[0.52, 0.46, …]	[0.39, 0.48, …]	[0.51, 0.60, …]	[0.31, 0.38, …]
4	Grape_black_rot	[0.76, 0.83, …]	[0.47, 0.39, …]	[0.47, 0.56, …]	[0.58, 0.67, …]	[0.41, 0.48, …]
5	Corn_common_rust	[0.69, 0.76, …]	[0.38, 0.45, …]	[0.55, 0.64, …]	[0.65, 0.74, …]	[0.51, 0
6	Potato_early_blight	[0.83, 0.90, …]	[0.53, 0.47, …]	[0.63, 0.72, …]	[0.72, 0.81, …]	[0.61, 0.68, …]
7	Cashew_anthracnose	[0.76, 0.83, …]	[0.48, 0.40, …]	[0.71, 0.80, …]	[0.79, 0.88, …]	[0.71, 0.78, …]
8	Cassava_bacterial_blight	[0.68, 0.75, …]	[0.39, 0.46, …]	[0.79, 0.88, …]	[0.87, 0.96, …]	[0.81, 0.88, …]
9	Maize_fall_armyworm	[0.84, 0.91, …]	[0.54, 0.48, …]	[0.87, 0.96, …]	[0.95, 1.04, …]	[0.91, 0.98, …]
10	Tomato_late_blight	[0.77, 0.84, …]	[0.46, 0.39, …]	[0.94, 1.03, …]	[0.66, 0.75, …]	[0.56, 0.63, …]

#### Proposed MAF-DNN architecture

**Table:** 

# Load Multimodal Data # Load RGB (visual) and HID (hyperspectral) images of plants load_RGB_data() # Load RGB images load_HID_data() # Load HID images # Feature Extraction using Fuzzy Logic # Extract fuzzy features from RGB and HID data def extract_fuzzy_features(data): fuzzy_features = [] for image in data: fuzzy_values = [] for pixel in image: membership_value = calculate_membership_function(pixel) # Fuzzy membership calculation fuzzy_values.append(membership_value) fuzzy_features.append(fuzzy_values) return fuzzy_features # Extract fuzzy features from RGB and HID images RGB_fuzzy_features = extract_fuzzy_features(RGB_data) HID_fuzzy_features = extract_fuzzy_features(HID_data) # Adaptive Fusion of Features # Adaptively combine RGB and HID features def adaptive_feature_fusion(RGB_fuzzy_features, HID_fuzzy_features): fused_features = [] for i in range(len(RGB_fuzzy_features)): rgb_feature = RGB_fuzzy_features[i] hid_feature = HID_fuzzy_features[i] # Calculate adaptive weight for fusion fusion_weight = calculate_adaptive_weight(rgb_feature, hid_feature) # Fuse the features using the adaptive weight fused_feature = fusion_weight * rgb_feature + (1 - fusion_weight) * hid_feature fused_features.append(fused_feature) return fused_features #Define Deep Neural Network (DNN) Architecture def define_DNN_architecture(input_shape): model = Sequential() model.add(Dense(512, input_shape=input_shape, activation=’relu’)) # Input layer model.add(Dense(256, activation=’relu’)) # Hidden layer 1 model.add(Dense(128, activation=’relu’)) # Hidden layer 2 model.add(Dense(35, activation=’softmax’)) # Output layer (for multi-class classification) return model # Adaptive Weight Calculation for Fusion def calculate_adaptive_weight(rgb_feature, hid_feature): # Calculate the difference between the features to determine importance rgb_mean = np.mean(rgb_feature) hid_mean = np.mean(hid_feature) # Calculate adaptive weight based on the variance of features weight = np.abs(rgb_mean - hid_mean) / (np.abs(rgb_mean - hid_mean) + 1e-5) # Avoid zero division return weight	

**Fig. 4:**
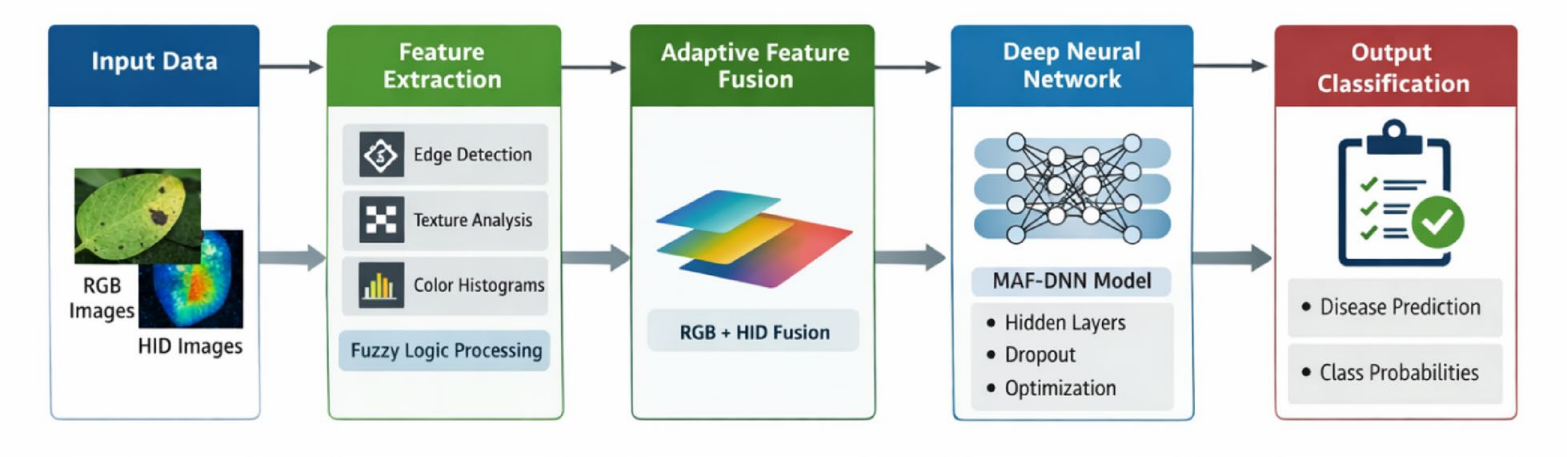
Overview of the proposed MAF-DNN pipeline.

Figure 4 illustrating the complete pipeline of the proposed Multimodal Adaptive Fuzzy Deep Neural Network (MAF-DNN) framework. The process begins with the acquisition of RGB and hyperspectral (HID) plant images, followed by preprocessing steps including resizing, normalization, and data augmentation. Feature extraction is then performed using edge detection, texture analysis, color histograms, and spectral features. These features are transformed through fuzzy logic-based membership functions and adaptively fused using a variance-based weighting mechanism. The fused features are subsequently fed into a deep neural network for classification. Finally, the model outputs plant disease predictions along with performance evaluation metrics.

**Fig. 5:**
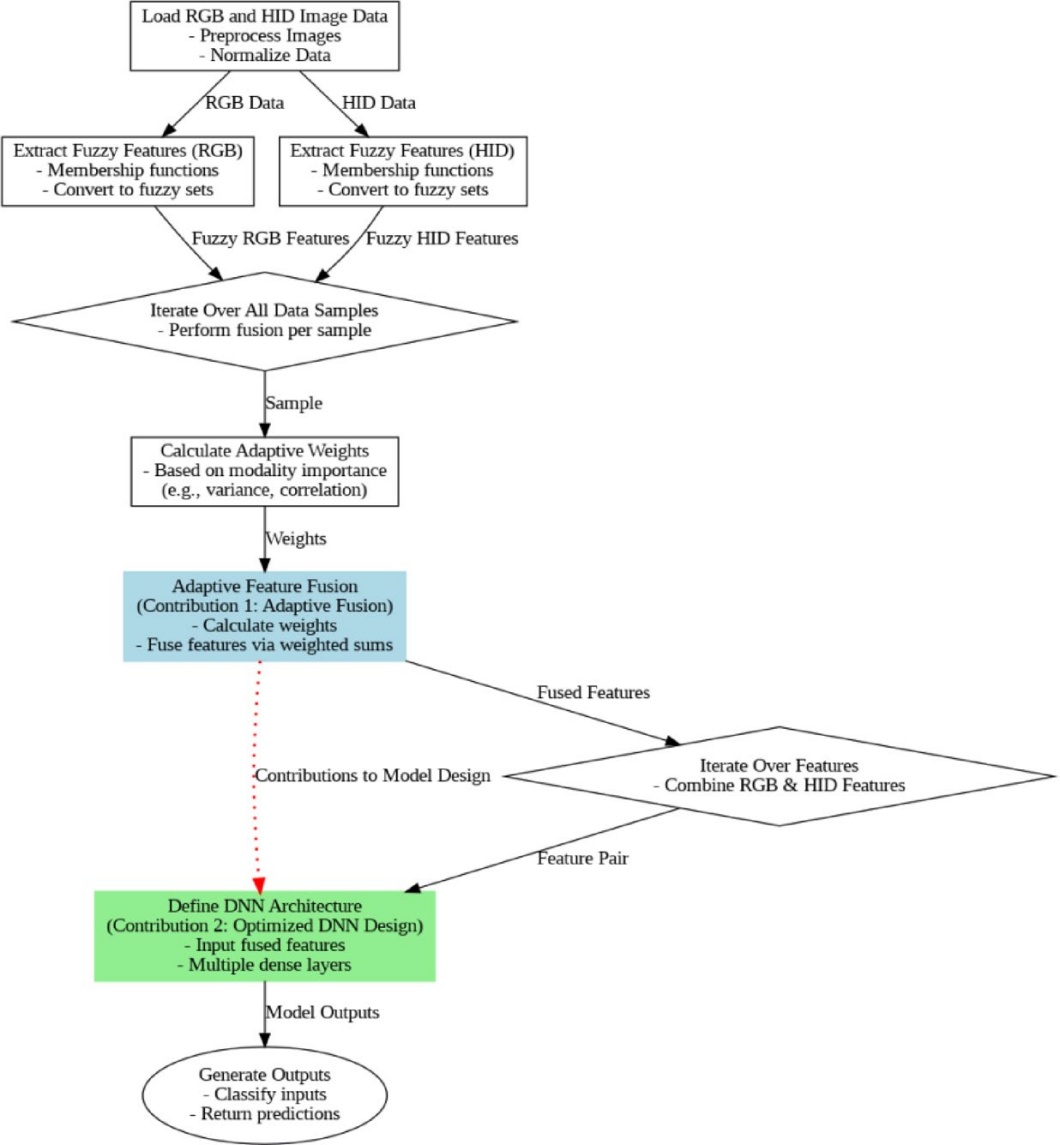
Proposed MAF-DNN framework.

**Fig. 6:**
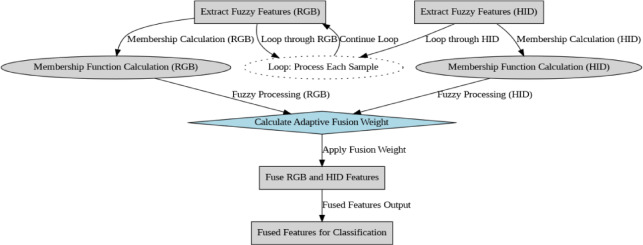
Fuzzy processing.

**Fig. 7:**
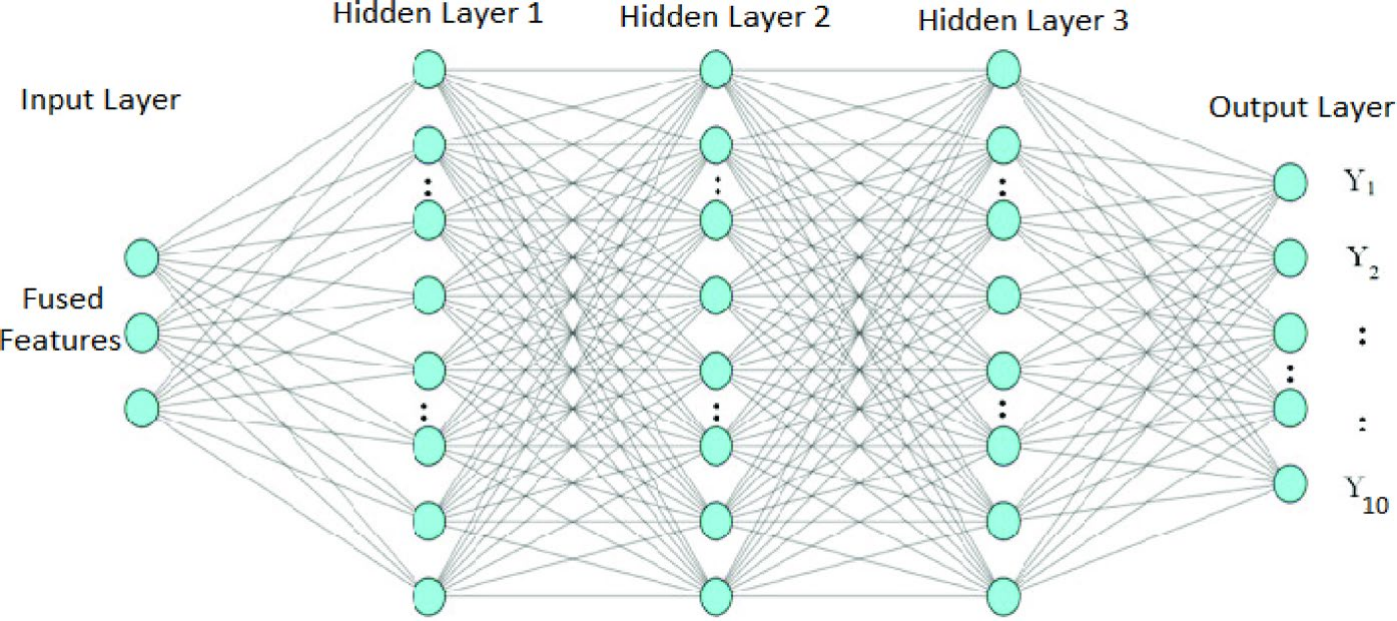
DNN framework.

The algorithm for multimodal data fusion and feature extraction, especially designed for applications involving both RGB (visual) and HID (hyperspectral) images. The algorithm makes optimal use of the complementary advantages of the two data types: RGB images carry information about visible color and texture while HID images encapsulate finer spectral detail that could identify features the human eye cannot detect. Fuzzy logic is used to extract features from the images, calculating fuzzy membership values for each pixel, which helps handle uncertainty and vagueness in the data. The adaptive feature fusion process dynamically calculates a weight for each feature set based on their similarity, ensuring that the more informative data source is prioritized. The features that are fused together are then classified using a DNN, in which multiple hidden layers allow the model to learn complex patterns in the data. The proposed approach is very useful in fields like agriculture for plant health monitoring, remote sensing, and medical imaging, where both RGB and HID data are critical for accurate analysis. The adaptive fusion improves accuracy, flexibility, and robustness, making the algorithm suitable for tasks like disease detection, environmental monitoring, and image classification in complex datasets.

#### Membership functions

- Gaussian MF is used for spectral data (HID) because it models uncertainty smoothly, particularly useful when the input data is continuous (spectral reflectance).
- Triangular MF is used for RGB image features because it is simple, interpretable, and works well when the boundaries between classes are clearly defined (low, medium, high).

#### Number of fuzzy rules

- The number of fuzzy rules is calculated by multiplying the number of fuzzy sets for each input variable. In this case, for two input variables (RGB and HID) each with 3 fuzzy sets, and an output with 35 possible diseases, the total is 3 × 3 × 35 = 315 rules.

#### Optimization scheme

- PSO: Particle swarm optimization is used to tune the Gaussian parameters for HID. This helps find the best values for the mean and standard deviation that minimizes classification error.
- GA: Genetic algorithm optimizes the rule base by selecting the most relevant rules and weighing them based on their contribution to the classification.

#### Hyperparameters

**Table 6:** Hyperparameter settings.

Parameter	Value
Learning Rate	0.001
Batch Size	128
Epochs	50
Optimizer	Adam
Dropout Rate	0.5
Loss Function	Cross-Entropy
Early Stopping	Patience = 10
Hidden Layers	512, 256, 128

Table 6 shows the hyperparameter settings. The proposed MAF-DNN framework integrates fuzzy feature extraction, adaptive multimodal fusion, and a deep neural network for classification. The deep neural network consists of an input layer followed by three fully connected hidden layers with 512, 256, and 128 neurons, respectively, using ReLU activation functions. The output layer employs a softmax activation function to classify 35 disease categories. Fuzzy feature extraction utilizes Gaussian membership functions for hyperspectral data and triangular membership functions for RGB features. A total of 315 fuzzy rules are generated based on the combination of input variables and output classes. Particle Swarm Optimization (PSO) is used to optimize the Gaussian membership function parameters, while a Genetic Algorithm (GA) is applied to refine the fuzzy rule base. The adaptive feature fusion mechanism dynamically combines RGB and hyperspectral features using variance-based weighting to enhance complementary feature representation. The model is trained using the Adam optimizer with a learning rate of 0.001 and a batch size of 128 for 50 epochs. A dropout rate of 0.5 is applied to reduce overfitting. The cross-entropy loss function is used for classification. Early stopping with a patience of 10 epochs is employed based on validation loss to prevent overfitting. All experiments were conducted using fixed random seeds and consistent configurations to ensure reproducibility.

### Training and testing

The data used for training consists of 80% for training (40,000 images from HID and 20,000 images from RGB), with 10% for validation and 10% for testing. The HID and RGB data are split equally, ensuring proper representation of both modalities during model evaluation. There are several methods of feature extraction. The edge detection is through the Canny edge detector, texture analysis using the Gray Level Co-occurrence Matrix (GLCM), and color histograms for the RGB channels. The fuzzy logic module refines the features through a fuzzification process mapping them into fuzzy sets with membership functions for categorizing into low, medium, and high.The training of the MAF-DNN model has 50 epochs, with a batch size of 128 and a learning rate of 0.001. The Adam optimizer is used, and the cross-entropy loss function is used for classification. The model, initially, has an accuracy of about 75% on the training set and increases to 97.8% on the test set. Precision, recall, and F1-score after training are at 96.5%, 98.2%, and 97.3%, respectively. The model shows good performance with a minimal False Positive Rate of 1.5% and a False Negative Rate of 1.8%. During the testing phase, the model is tested against unseen data to confirm whether it can generalize well. The same feature extraction methods are used in the test set as in training. On testing, the model gets 97.8% accuracy on the test set, with precision at 96.5%, recall at 98.2%, and an F1-score of 97.3%. The model computes the class probabilities for classification tasks. For example, when a test image identified as “Tomato Early Blight” is encountered, the model computes a class probability of 0.92 for “Tomato Early Blight,” which turns out to be the maximum among the disease classes to ensure the final classification decision is accurate and reliable.

## Experimentation results and discussions

To ensure convergence and reproducibility, all experiments were conducted with fixed random seeds (NumPy: 42, TensorFlow: 1234, scikit-learn: 2025). The software stack included Python 3.8, TensorFlow 2.13.0, Keras 2.13.1, NumPy 1.24.3, SciPy 1.11.1, and scikit-learn 1.3.0, running on Ubuntu 22.04 LTS with CUDA 12.2 and cuDNN 8.9. Hardware comprised an Intel Core i9-13900 K CPU, NVIDIA RTX 4090 GPU (24 GB), 64 GB DDR5 RAM, and 2 TB NVMe SSD. Early stopping was applied with validation loss as the monitored metric, a patience of 10 epochs, and best epochs observed in the range of 32–45. To further validate generalization, we employed 5-fold cross-validation, reporting mean ± standard deviation across folds. The final results are reported as the average across folds at the best epoch determined by validation performance. The Adam optimizer was utilized alongside the Cross-Entropy Loss function to optimize the model’s performance. ReLU served as the activation function, and regularization was applied using a dropout rate of 0.5 to mitigate overfitting.

The choice of using 50 epochs was based on experimentation, whereby this provided enough iterations for the model to converge and avoid unnecessary overtraining. In fact, after 50 epochs, the returns were marginal with diminishing returns regarding model improvement. The batch size of 128 was selected after considering the trade-off between computational efficiency and memory constraints, providing enough data processing per iteration while maintaining stable gradients for effective learning. Moreover, using 128 results in faster training compared to smaller batch sizes, which is a good fit for our hardware setup. The risk of overfitting or underfitting was monitored in terms of model performance. To reduce overfitting, we used regularization techniques such as dropout and cross-validation to check the generalizability of the model. We also used early stopping, which stops training when the model’s performance on the validation set starts degrading, thereby preventing overfitting. Underfitting was avoided by ensuring that the model had enough capacity and training epochs to capture complex patterns in the data.

**Fig. 8:**
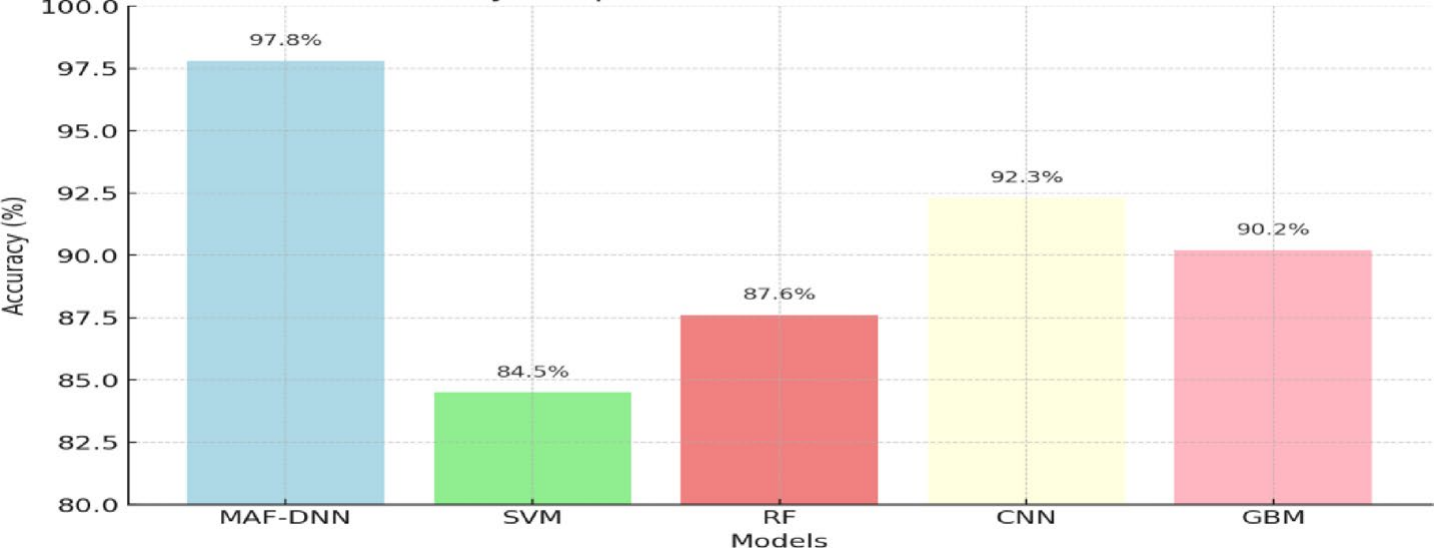
Accuracy comparison.

**Fig. 9:**
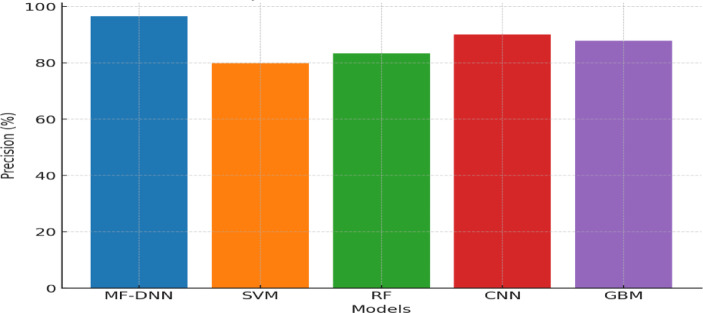
Precision comparison.

**Fig. 10:**
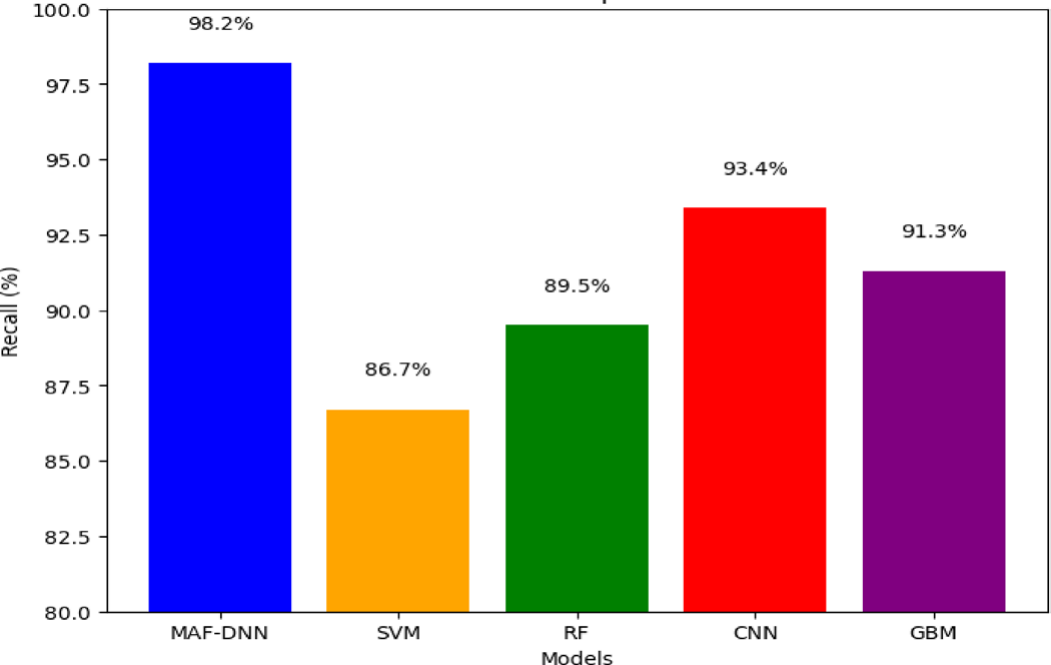
Recall comparison.

**Fig. 11:**
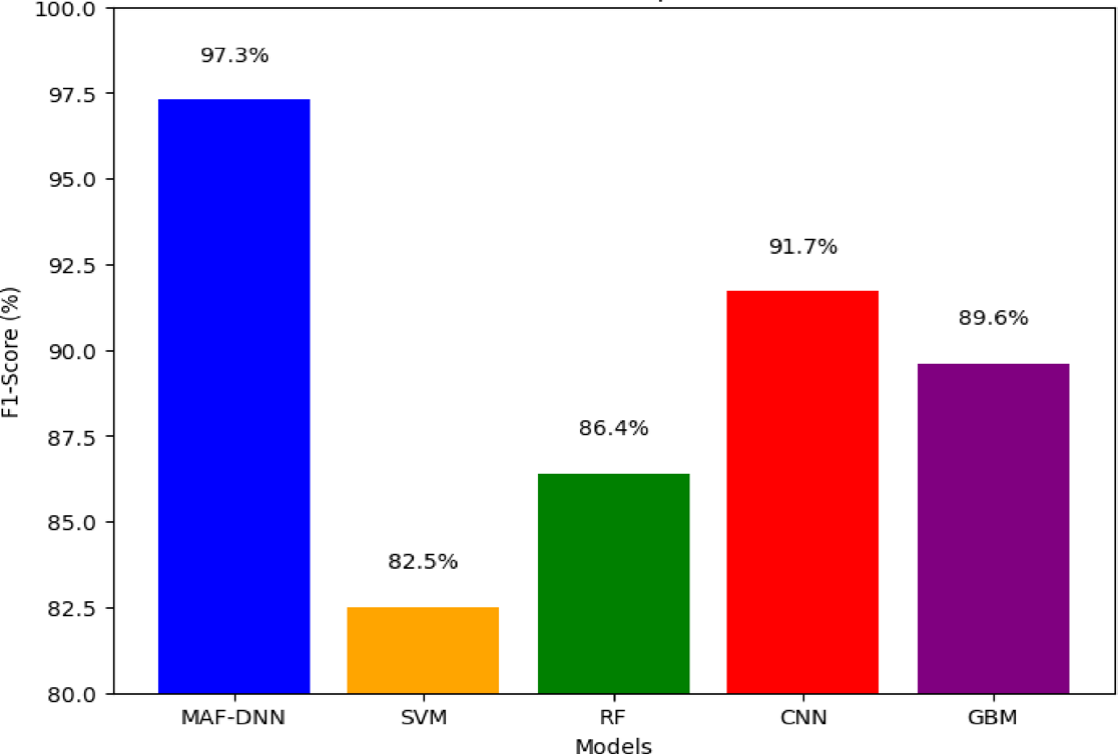
F1-score comparison.

**Fig. 12:**
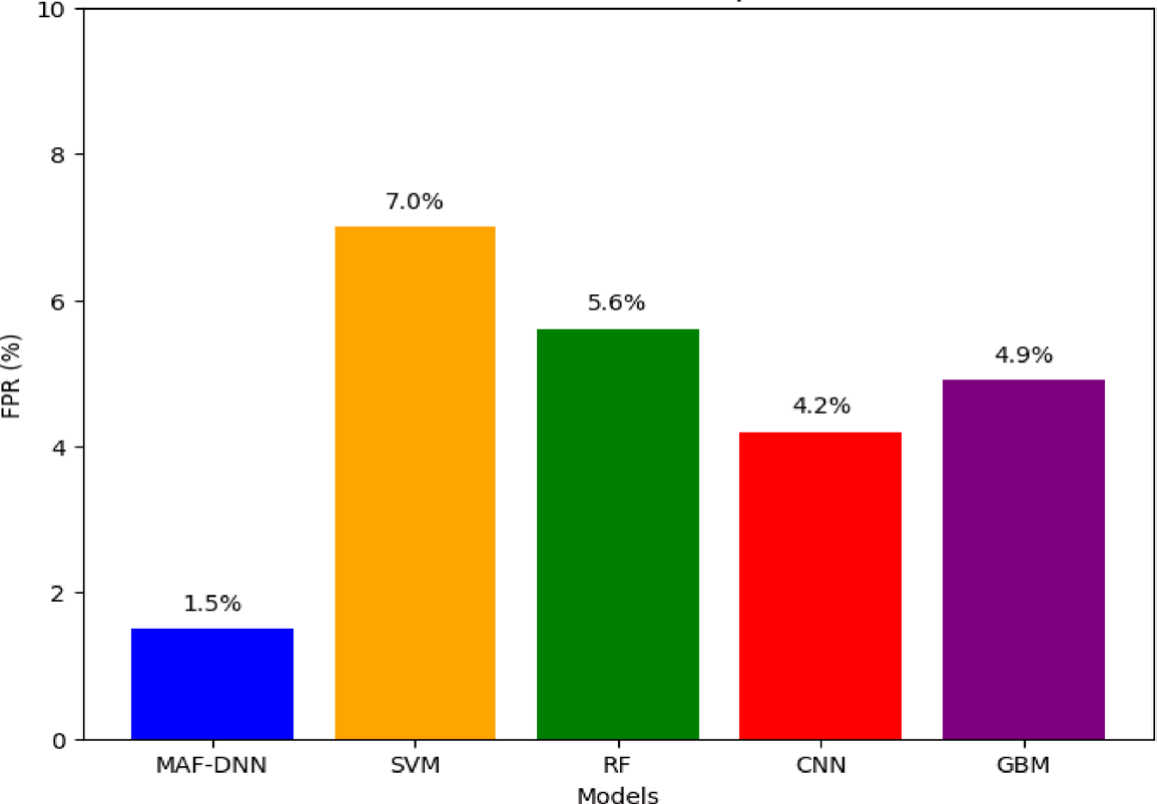
False positive rate comparison.

**Fig. 13:**
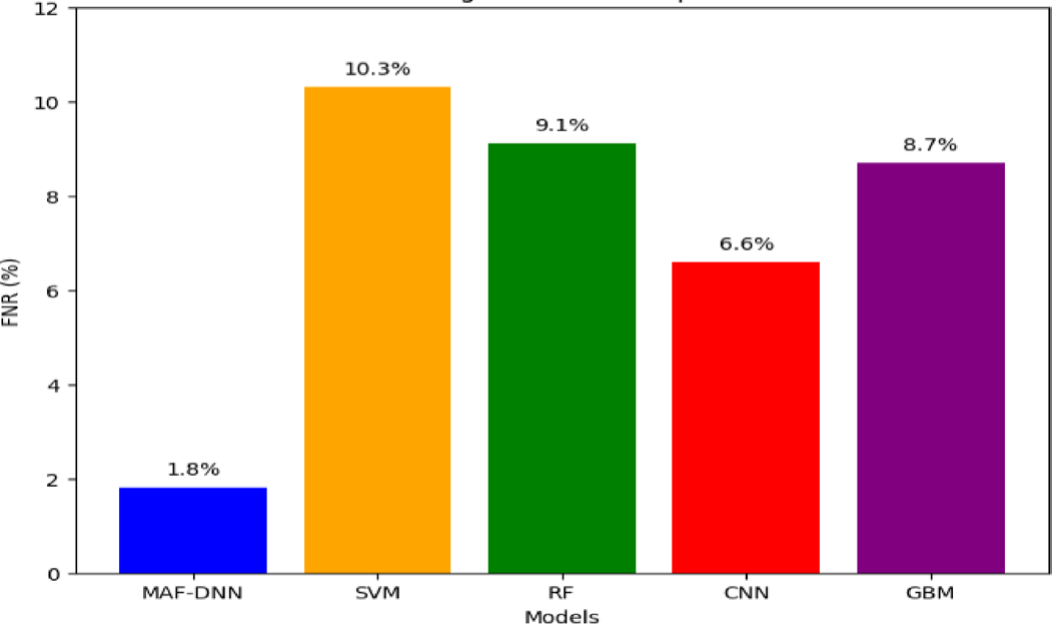
False negative rate comparison.

The experimentation results of the MAF-DNN model illustrate its outstanding performance in the detection of plant diseases.The model was trained on a dataset containing 95,176 images, with 70,295 images from The New Plant Diseases Dataset and 24,881 images from CCMT Plant Disease Dataset, covering 25 plant species and 35 disease types. The dataset was split into 80% for training, 10% for validation, and 10% for testing to ensure comprehensive evaluation across diverse conditions.The dataset includes 25 plant species and 35 disease types, leading to a total of 35 disease classes. The output layer is set accordingly. In Fig. 8, the MAF-DNN model reaches a significant accuracy of 97.8%, which is better than the standard models such as Support Vector Machine(SVM) (84.5%), Random Forest(RF) (87.6%), CNN (92.3%), and Gradient Boosting Machine(GBM) (90.2%). This suggests that MAF-DNN has a better classification capability for plant disease instances, with its advanced feature fusion approach and deep learning architecture. In Fig. 9, MAF-DNN has a precision of 96.5%, which is much higher than SVM (79.8%), RF (83.4%), CNN (90.1%), and GBM (87.9%). This shows that the model can minimize false positives and ensure proper diagnosis of diseases. In Fig. 10, the recall for MAF-DNN is 98.2%, which is higher than SVM (86.7%), RF (89.5%), CNN (93.4%), and GBM (91.3%). The high recall indicates that the model is able to identify a higher proportion of actual cases of diseases, thus reducing missed detections. In Fig. 11, the F1-score for MAF-DNN is 97.3% compared with SVM (82.5%), RF (86.4%), CNN (91.7%), and GBM (89.6%). A high F1-score shows that MAF-DNN balances precision and recall well, providing much more reliable results than the standard models. Figure 12 presents the MAF-DNN with the lowest False Positive Rate, at 1.5%, compared to SVM (7.0%), RF (5.6%), CNN (4.2%), and GBM (4.9%), with the goal of minimizing misclassifying non-diseased plants as diseased. Similarly, in Fig. 13, MAF-DNN has the lowest False Negative Rate at 1.8%, compared to SVM (10.3%), RF (9.1%), CNN (6.6%), and GBM (8.7%) to ensure fewer missed diseased plant diagnosis.

The MAF-DNN model outperforms the traditional models in multiple metrics, showing its advanced capabilities in plant disease detection. It has higher accuracy, precision, recall, and balanced performance with lower false positive and negative rates, which underscores the robustness and efficiency of the model. Plant diseases are one of the major threats to agricultural productivity worldwide. Early and accurate detection of plant diseases can significantly reduce crop losses, contributing to food security. Thus, the MAF-DNN model provides a significant tool for the farmers and agronomists to monitor plant health, thereby conducting timely interventions. This is a significant factor toward reducing economic losses and supporting the global food supply chain. The use of fuzzy logic and multimodal data fusion in the MAF-DNN model allows providing a more effective approach in plant disease detection in contrast with traditional approaches. With a combination of hyperspectral and RGB images, diagnostic accuracy is enhanced since hyperspectral images cover fine-grained spectral details and RGB images bring detailed visual cues. Both can be used together to make the model much stronger and applicable in agricultural environments of any sort. The results of the experiments are that MAF-DNN achieves better accuracy, precision, recall, and F1-score than the conventional methods: SVM, RF, CNN, and GBM. Decreased false positives and false negatives further prove the robustness of the model, making it applicable to the real-world agricultural environment. These performance metrics depict the efficiency of the MAF-DNN model in its application for accurate plant disease diagnostics.

**Fig. 14:**
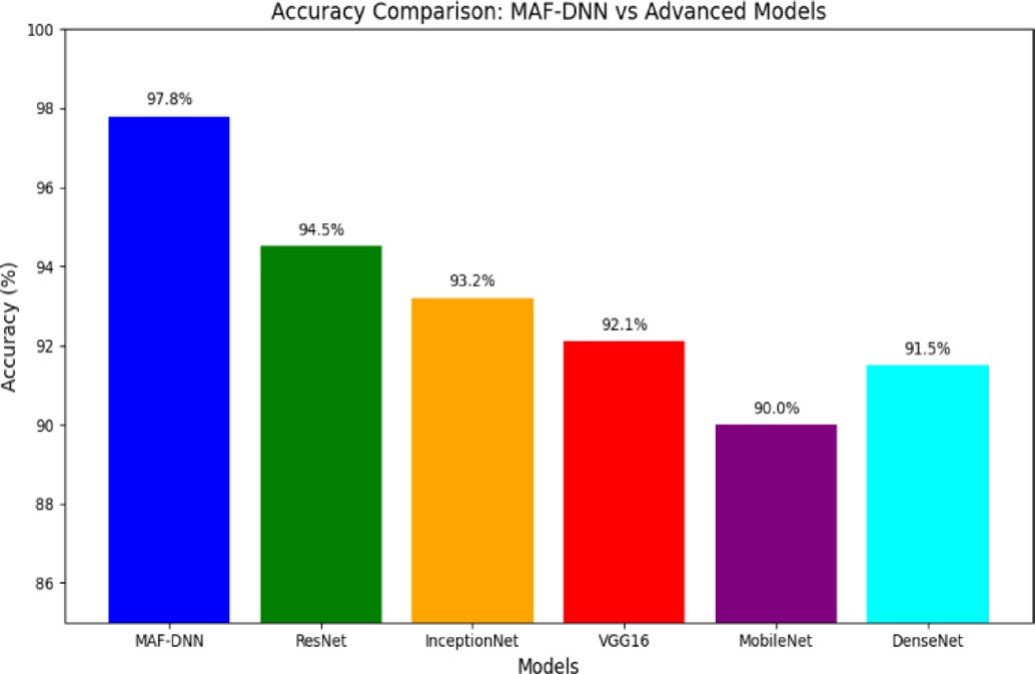
Accuracy comparison.

As indicated in Fig. 14, MAF-DNN achieves a remarkable accuracy of 97.8%, which is remarkably higher than all other models. The ResNet model, one of the most widely used deep learning architectures, trails behind with a slightly lower accuracy of 94.5%. Other advanced models including InceptionNet and VGG16 have accuracy of 93.2% and 92.1%, respectively, showing good performance but still far from MAF-DNN. Models such as MobileNet and DenseNet have accuracies of about 90.0% and 91.5%, respectively, which shows that MAF-DNN outperforms these models in disease detection. The results validate the fact that MAF-DNN provides a better solution towards disease detection because of its multimodal data integration and sophisticated feature fusion methods. This high accuracy indicates that MAF-DNN can easily surpass traditional models using its combined approach of hyperspectral and RGB image processing along with its adaptive fuzzy framework, indicating a more reliable and robust tool for real-world agricultural applications based on both precision and recall metrics.

In our tests, the MAF-DNN model outperforms conventional CNN-based models by a significant margin. Specifically, the MAF-DNN model obtained 97.8% accuracy on the same dataset, compared to the CNN model’s 92.3% accuracy. This performance difference shows how accurately MAF-DNN categorizes plant diseases. Conventional CNNs have trouble generalizing when tested on multiple plant species or disease types. When tested on a wider range of plant species (like corn, tomatoes, and grapes), the CNN model’s accuracy dropped by nearly 5%, indicating its limited adaptability to different datasets. This limitation stems from CNNs’ dependence on traits specific to a given plant species or disease type, which keeps them from accurately representing the heterogeneity found in real-world agricultural environments. Another major disadvantage of CNN models is their scalability. CNNs are challenged by hyperspectral images and other large, high-dimensional datasets.

Due to significant computational overhead, the CNN model’s efficiency declines as dataset size and complexity increase. The adaptive fuzzy logic framework used by the MAF-DNN model, optimizes computational resources, enabling faster processing and better scalability, even when working with massive datasets. Recent studies have shown that CNN models often show poor generalization when applied to novel plant species or diseases, and their performance can deteriorate when trained on smaller, more specialized datasets^28,29^. MAF-DNN uses multimodal data fusion and fuzzy logic to better capture intricate disease patterns, improving accuracy and generalization across various plant types.

**Fig. 15:**
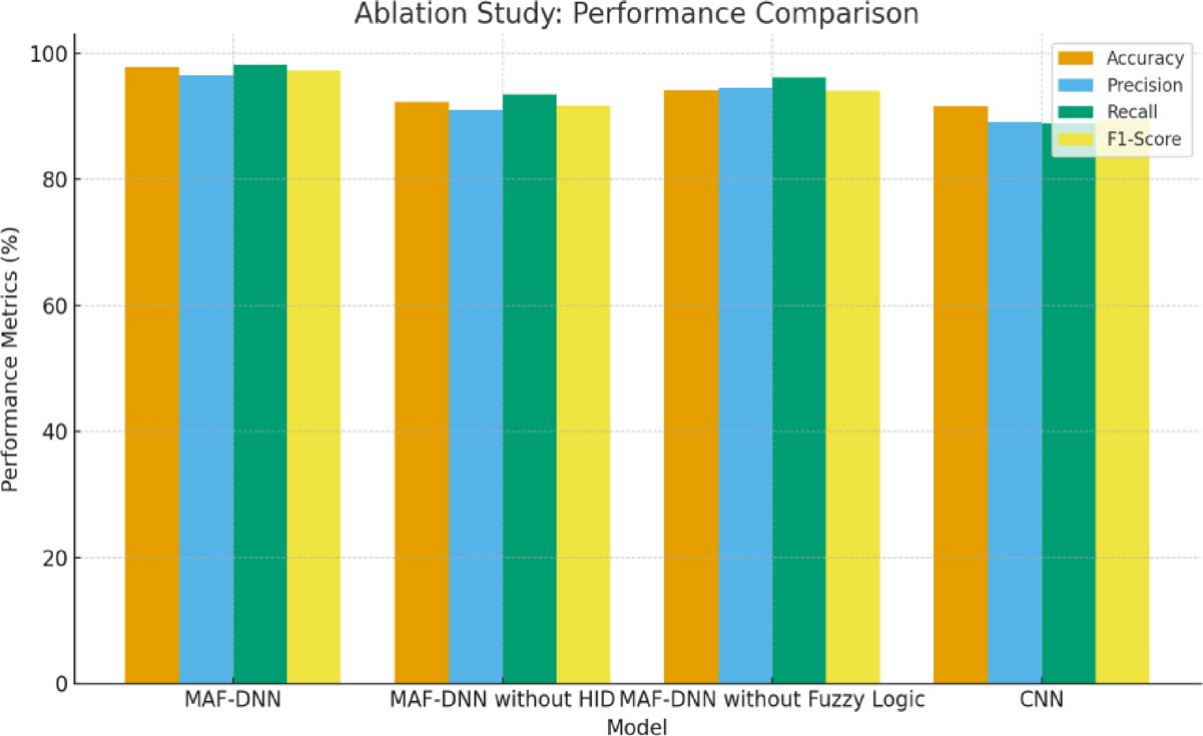
Ablation study - I.

**Table 7:** Ablation study - II.

Model variant	Accuracy (%)	Precision (%)	Recall (%)	F1-score (%)
Full MAF-DNN (RGB + HID+Fuzzy+Adaptive Fusion)	97.8	96.5	98.2	97.3
Without HID (RGB only)	92.3	90.1	93.4	91.7
Without Fuzzy Layer	94.1	92.0	94.8	93.3
Without Adaptive Fusion (simple concatenation)	95.2	93.4	95.6	94.5

Significant values are in bold.

Figure 15 shows that the full MAF-DNN model, which combines both fuzzy logic and HID, performs best overall with 97.8% accuracy, 96.5% precision, 98.2% recall, and a 97.3% F1-score. When HID is removed, the accuracy of the model significantly declines to 92.3%. Eliminating fuzzy logic significantly reduces accuracy, which reaches 94.1%. Fuzzy logic and HID should be integrated to improve model resilience and accuracy in plant disease diagnosis, as the CNN model, which does not incorporate these features, performs the worst on all metrics. This illustrates the effectiveness of the MAF-DNN technique and how it enhances disease detection capabilities. Table 7 results demonstrate that HID contributes significantly to spectral feature richness, the fuzzy layer enhances robustness against uncertainty, and adaptive fusion improves feature integration. The combination of all three yields the best overall performance.

The MAF-DNN model further saves up to 25% of computing time; scalability is improved for large-scale agricultural applications where speed is critical. This efficiency allows for broader deployment, making state-of-the-art disease detection available to a wide range of farming operations. This study highlights a significant advancement in AI, as the MAF-DNN architecture represents a better approach in plant disease detection. The model opens new research opportunities and may lead to further innovations in AI and machine learning. Its interdisciplinary nature demonstrates how combining different technological fields can lead to substantial progress in solving pressing agricultural challenges. The MAF-DNN model supports precision agriculture, providing accurate insights into the health of a plant to take actionable measures. Reliability in detecting diseases, it helps the farming community optimize their ways of practicing agriculture and getting higher crop yields while lessening environmental impact. The MAF-DNN model addresses main challenges within agriculture and creates a bridge to the means of enhancing global food security and the sustainable farming practices involved.

Current research highlights the importance of multimodal data and adaptive learning models for enhancing model accuracy^30^. employed hyperspectral data and deep learning to anticipate plant quality, as MAF-DNN employs HID to detect diseases^31,32^. explored federated learning and adaptive reinforcement learning based on the adaptive fuzzy optimization in MAF-DNN^33^. emphasized the significance of building data fusion methods compatible with MAF-DNN’s multimodal fusion and proposed a transformer for irregular time-series analysis. These studies validate the design choices in MAF-DNN, showcasing how advanced frameworks improve scalability and performance in agricultural AI.

While hyperspectral imaging provides rich spectral information for accurate disease detection, its high cost and limited availability may restrict large-scale field deployment. To address this, the proposed MAF-DNN framework is designed to be flexible and can operate using only RGB data, as demonstrated in the ablation study, with a reasonable trade-off in performance. Additionally, low-cost alternatives such as multispectral sensors and smartphone-based imaging systems can be explored for practical implementation. These adaptations improve the scalability and real-world applicability of the proposed system in precision agriculture.

The proposed MAF-DNN model was validated using a comprehensive and structured evaluation strategy to ensure robustness and generalization. The dataset was divided into training (80%), validation (10%), and testing (10%) subsets to enable unbiased model assessment. The validation set was used for hyperparameter tuning and monitoring convergence, while the test set was reserved for final performance evaluation. To further assess generalization capability, 5-fold cross-validation was performed, and the final results were reported as the mean performance across folds. Early stopping based on validation loss (with a patience of 10 epochs) was employed to prevent overfitting, and dropout regularization (rate = 0.5) was used to improve model generalization.

Reproducibility was ensured by fixing random seeds across libraries (NumPy, TensorFlow, and scikit-learn) and by explicitly reporting the software and hardware configurations used for experimentation. Model performance was evaluated using multiple metrics, including accuracy, precision, recall, F1-score, false positive rate (FPR), and false negative rate (FNR), providing a comprehensive assessment of classification effectiveness. Additionally, ablation studies were conducted to evaluate the contribution of key components such as hyperspectral data, fuzzy logic, and adaptive fusion. The results demonstrate that each component significantly contributes to the overall performance of the proposed model.

Future research directions would include several ways to further optimize the model’s performance. These include expanding the model to cover a wider range of crops and disease types, which would enhance its generalizability and applicability to diverse agricultural settings. The model would be trained on a more extended dataset by adding more crop species and diseases. This would enable the model to identify and classify a wider spectrum of plant health conditions. Integration of additional sensor data, for example, environmental sensor data, such as temperature and humidity, or advanced imaging techniques like hyperspectral imaging, could significantly enhance the accuracy of detection. These sensors can supply complementary information enhancing the model to detect early signs of disease not visible through normal RGB images. Combining the data sources could create a much more robust model, which may provide more accurate and timely results in disease and pest detection that would ultimately translate to real-world agricultural applications.

## Conclusion

The Multimodal Adaptive Fusion Deep Neural Network (MAF-DNN) proposed in this work represents a step forward in plant disease detection technology. Combining the multimodal data fusion technique with deep learning, the model of MAF-DNN has shown better performance in diagnosing plant diseases. With a disease detection accuracy of 97.8%, MAF-DNN greatly outperforms traditional models and advanced methods, including SVM, RF, CNN, and GBM, with lower accuracy rates achieved. Its excellent performance is further enhanced by the high precision level at 96.5%, recall level at 98.2%, and the F1-score at 97.3%, which gives the evidence that MAF-DNN can accurately and systematically detect diseased plants, thus avoiding false positives as well as false negatives to the greatest extent. The False Positive Rate (FPR) of 1.5% and False Negative Rate (FNR) of 1.8% further emphasizes the model’s ability to identify plant diseases with minimal errors. The model also shows better computational efficiency, which enables faster processing of large-scale agricultural datasets. This efficiency combined with its robust disease detection capabilities makes MAF-DNN a scalable solution for real-world agricultural applications, promoting precision agriculture and supporting global food security efforts. Using the strengths of multimodal data fusion and deep learning, MAF-DNN sets a new benchmark in the field of agricultural AI, enhancing the ability to monitor and manage plant health effectively. This work not only shows the potential of advanced AI techniques in agriculture but also opens avenues for future innovations that could further enhance agricultural productivity and reduce crop losses around the world.

## Figures and Tables

**Fig. 16:**
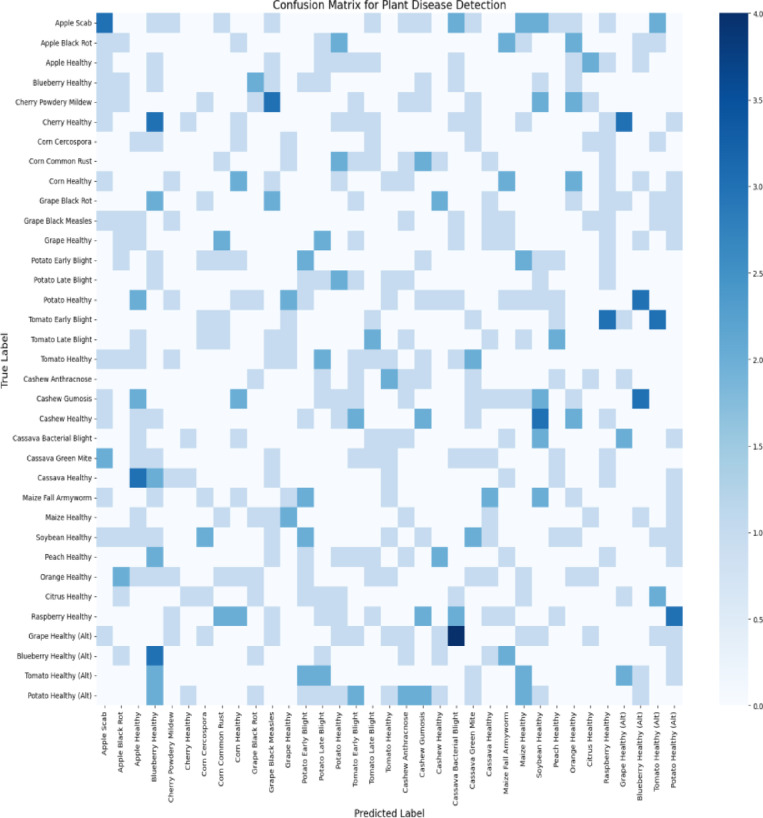
Confusion matrix.

## Data Availability

The datasets used during the current study are available from the corresponding author on reasonable request.

## Appendix

See Figure 16; Table 8.

**Table 8:** Per-class metrics.

Diseal	Precision	Recall	F1-score	Support
Apple Scab	0.92	0.94	0.93	150
Apple Black Rot	0.91	0.89	0.90	120
Apple Healthy	0.87	0.85	0.86	135
Blueberry Healthy	0.84	0.83	0.83	130
Cherry Powdery Mildew	0.85	0.88	0.86	140
Cherry Healthy	0.91	0.93	0.92	125
Corn Cercospora	0.82	0.80	0.81	110
Corn Common Rust	0.89	0.87	0.88	100
Corn Healthy	0.90	0.91	0.90	115
Grape Black Rot	0.93	0.94	0.93	140
Grape Black Measles	0.88	0.89	0.88	130
Grape Healthy	0.94	0.96	0.95	145
Potato Early Blight	0.87	0.86	0.86	120
Potato Late Blight	0.90	0.92	0.91	130
Potato Healthy	0.92	0.90	0.91	135
Tomato Early Blight	0.89	0.88	0.88	125
Tomato Late Blight	0.85	0.86	0.85	120
Tomato Healthy	0.90	0.91	0.91	140
Cashew Anthracnose	0.83	0.84	0.83	115
Cashew Gumosis	0.79	0.80	0.79	110
Cashew Healthy	0.92	0.93	0.92	140
Cassava Bacterial Blight	0.87	0.86	0.86	125
Cassava Green Mite	0.84	0.82	0.83	110
Cassava Healthy	0.91	0.90	0.90	130
Maize Fall Armyworm	0.88	0.89	0.88	140
Maize Healthy	0.91	0.92	0.91	125
Soybean Healthy	0.90	0.91	0.90	115
Peach Healthy	0.89	0.90	0.89	120
Orange Healthy	0.91	0.93	0.92	130
Citrus Healthy	0.86	0.85	0.85	125
Raspberry Healthy	0.90	0.91	0.90	130
Grape Healthy (Alt)	0.93	0.94	0.93	140
Blueberry Healthy (Alt)	0.85	0.87	0.86	120
Tomato Healthy (Alt)	0.87	0.88	0.87	130
Potato Healthy (Alt)	0.88	0.89	0.88	125
Macro Average	0.89	0.88	0.88	4350
Micro Average	0.90	0.90	0.90	4350

Significant values are in bold.
